# mSphere of Influence: Microbiome-Associated Phenotypes Are Modifiable

**DOI:** 10.1128/mSphere.00508-20

**Published:** 2020-07-01

**Authors:** Michael H. Woodworth

**Affiliations:** a Emory University School of Medicine, Department of Medicine, Division of Infectious Disease, Atlanta, Georgia, USA

**Keywords:** fecal microbiota transplant, kwashiorkor, malnutrition, microbiome

## Abstract

Michael Woodworth focuses on translational microbiome therapeutic research. In this mSphere of Influence article, he reflects on how “Gut microbiomes of Malawian twin pairs discordant for kwashiorkor*”* by Michelle Smith et al. (M. I. Smith, T. Yatsunenko, M. J. Manary, I. Trehan, et al., Science 339:548–554, 2013, https://doi.org/10.1126/science.1229000) made an impact on him by revealing the causal influence of microbial communities in the development of severe malnutrition.

## COMMENTARY

In 2012, Kathy Kirkland gave our fourth-year medical school class a revelatory talk on the use of fecal microbiota transplantation (FMT) to treat *Clostridioides* (formerly *Clostridium*) *difficile* infection. As we learned about the therapeutic potential of feces, which had not been discussed in our pharmacology lectures, the energy in the room was palpable. The direct transfer of microbial communities to treat a disease that is strongly associated with antibiotics felt both rebellious and completely intuitive. Not long after this lecture, the paper “Gut microbiomes of Malawian twin pairs discordant for kwashiorkor” by Michelle Smith et al. elegantly demonstrated the causal role of the intestinal microbiome in the development of another vexing and burdensome disease, kwashiorkor (1). Kwashiorkor is crippling to segments of developing societies. It is a particularly acute manifestation of extreme poverty that only transiently improves with nutritional supplementation. This paper was one of my first introductions to functional and compositional analysis of microbial communities as well as the feasibility of transplantation of human-associated microbial communities into germfree animal models. In addition, this paper expanded my sense of the scope of microbiome-associated phenotypes that could be amenable to microbiome therapies. As a result, this work had a major impact in developing my research focus on clinical and translational investigation of microbiome therapies like FMT.

Smith et al. performed a longitudinal comparative study of twin pairs in Malawi to examine the role of intestinal microbial communities in kwashiorkor (1). Prior work indicated that protein intake and oxidative stress did not adequately explain the development of kwashiorkor. This led the group to focus on a series of questions about the association of microbiome features and kwashiorkor. The authors selected 13 pairs of same-gender twins who were discordant for kwashiorkor and 9 pairs of same-gender twins who remained healthy by the World Health Organization definition of kwashiorkor and normal growth curves. Taxonomy was assigned using 16S and shotgun metagenomic sequence data, and functional annotation of metagenomic sequence data was performed with the Kyoto Encyclopedia of Genes and Genomes (KEGG). They demonstrated a blunting of normal age-related transitions in microbial community structure and function in co-twins with kwashiorkor compared to healthy co-twins and healthy weight concordant twin pairs. To more directly test causality, they transplanted fecal samples from the Malawian study participants into germfree C57BL/6J mice that were fed a typical Malawian diet punctuated by supplementation with ready-to-use therapeutic food. Remarkably, the mice who were fed a Malawian diet after transplantation with fecal microbiota from children with kwashiorkor lost >30% of their body weight over a 3-week period, while the mice receiving fecal microbiota from healthy children had stable or increased body weight (1). They also observed substantial efficiency of transplantation of species-level taxa and KEGG enzyme commission numbers (i.e., metabolic functional units) in the germfree mice. These mice with humanized intestinal microbiota were then used as a model to test taxonomic and metabolic dynamics in the context of the typical Malawian diet or ready-to-use therapeutic food. Taken together, observations from their Malawi cohort and mice with humanized intestinal microbiota suggested a causal role of microbial community configuration and diet in the development of kwashiorkor.

Among others around the same time, this paper inspired me to work toward developing a translational research program with human microbiome transplantation studies. It demonstrated an approach with metagenomic and metabolic analyses to determine ecological dynamics in human-associated microbial communities. In larger themes, this work also exemplified the importance of team science in clinical microbiome science and helped me prioritize skill sets to seek.

Bill Gates pointed to this study as evidence for his prediction that malnutrition will be solved in his Hawking Fellowship lecture in 2019 (https://www.gatesnotes.com/Health/Professor-Hawking-Fellowship-lecture). Since “Gut microbiomes of Malawian twin pairs discordant for kwashiorkor” was published, a substantial body of high-impact microbiome work has been done. Recently, Raman et al. working in the same group described an approach to identify sparse covarying taxa that might signal emergent groups of microbial communities associated with normal development and acute malnutrition (2). Many other groups are actively working to develop rational combinations of food and microbes to better treat malnutrition. Animal models with humanized microbiota are a widespread approach to improve understanding of the roles in normal development, immune function, and numerous diseases. Enthusiasm for therapeutic and diagnostic potential of microbiome research remains very high. However, as might be expected, the complexity of multi-omic studies that integrate metagenomic, transcriptomic, metabolomic, and other high-dimensional measures has also laid pitfalls for statistical bias and overstated conclusions. For example, one recent systematic review of 38 studies of human microbiota-associated rodents found that 95% reported a transfer of phenotypes from humans to the rodent model, which they interpreted as an indication of publication bias (3). They also found that 32/38 (84%) of the studies included used individual humanized animals as the unit of statistical analysis rather than the number of human donors. This approach presents a risk of pseudoreplication, which can inflate the chance of false-positive findings (3). Several other important challenges remain in defining microbiome-mediated mechanisms of health and disease. These challenges include the following. (i) Though less expensive than shotgun metagenomic sequencing, 16S rRNA sequence analysis does not measure fractions of viral, helminth, and fungal microbiota in disease states. Thus, the potential roles of these other taxa in health and disease have not been as extensively evaluated. (ii) Human microbiome interventional trials can be challenging to enroll in a timely fashion due to availability of FMT for outside of clinical trials despite lack of FDA approval and reservations about the aesthetics of FMT (4). (iii) Microbiome science lacks a validated definition, much less a diagnostic test for dysbiosis. (iv) Characterization and annotation of the dark matter of undefined taxa, microbial community structures, genes, gene products, and metabolites remain important barriers to clinical translation. More than anything, these limitations should be taken as signals that there is much good work to be done to translate understanding from microbiome studies to improve health and reduce suffering.

As an infectious disease physician, my research is focused on understanding the role of intestinal microbial ecology in colonization with antimicrobial-resistant bacteria and translating this knowledge to new treatments for patients. We are focused on identifying causal components of FMT that contribute to its observed clinical efficacy for eradicating colonization with antimicrobial-resistant bacteria. Our phase 1 clinical trials improve understanding of the safety and efficacy of FMT while also expanding access to this treatment in structured research protocols. I am still motivated by this paper’s demonstration of the malleability of phenotypes associated with microbial communities. Making antibiotic recommendations is one of my most frequent clinical activities. However, I am hopeful that within the span of my career, safe and effective microbiome therapies will be available to complement the use of antibiotics and potentially address severe malnutrition.
